# Mitochondria-Associated Endoplasmic Reticulum Membranes in the Pathogenesis of Type 2 Diabetes Mellitus

**DOI:** 10.3389/fcell.2020.571554

**Published:** 2020-10-20

**Authors:** Shanshan Yang, Ruixue Zhou, Caixia Zhang, Siyuan He, Zhiguang Su

**Affiliations:** Molecular Medicine Research Center and National Clinical Research Center for Geriatrics, West China Hospital, State Key Laboratory of Biotherapy, Sichuan University, Chengdu, China

**Keywords:** mitochondria, endoplasmic reticulum, mitochondria-associated endoplasmic reticulum membranes, insulin resistance, pancreatic β cell, diabetes

## Abstract

The endoplasmic reticulum (ER) and mitochondria are essential intracellular organelles that actively communicate via temporally and spatially formed contacts called mitochondria-associated membranes (MAMs). These mitochondria-ER contacts are not only necessary for the physiological function of the organelles and their coordination with each other, but they also control the intracellular lipid exchange, calcium signaling, cell survival, and homeostasis in cellular metabolism. Growing evidence strongly supports the role of the mitochondria-ER connection in the insulin resistance of peripheral tissues, pancreatic β cell dysfunction, and the consequent development of type 2 diabetes mellitus (T2DM). In this review, we summarize current advances in the understanding of the mitochondria-ER connection and specifically focus on addressing a new perspective of the alterations in mitochondria-ER communication in insulin signaling and β cell maintenance.

## Introduction

T2DM is the most common endocrine and metabolic disorder in humans, and it has increased dramatically in the past 20 years. In 2017, more than 451 million individuals worldwide were affected and it is estimated that the prevalence will reach 693 million by 2045 (Cho et al., 2018). T2DM is caused by peripheral insulin resistance and relative insulin deficiency owing to β cell dysfunction, where the development of disease is preceded by several years of insulin resistance (Eizirik et al., 2020). Therefore, better recognition of the molecular mechanisms involved in insulin resistance and β cell dysfunction is essential to develop preventive or therapeutic strategies. Intracellular organelles, such as mitochondria and ER, have emerged as critical regulators of insulin signaling and β cell function (Chen et al., 2019; Siasos et al., 2020), highlighting their importance in maintaining glucose homeostasis and controlling the pathophysiology of T2DM. Both ER and mitochondria membranes are closely apposed and form contact sites through protein-protein or protein-lipid tethering complexes, which are essential for maintaining fundamental cellular functions by exchanging lipids and calcium (Ca^2+^) between the two organelles (Lee and Min, 2018). The dysfunctional mitochondrial-ER connection may be a new mechanism underlying T2DM.

In this review, we introduce the architecture and biological functions of mitochondria-ER contacts and focus mainly on their involvement in glucose homeostasis, while highlighting recent findings on the role of ER-mitochondria miscommunication in the development of T2DM.

## Mitochondria-ER Contacts and the Architecture of Tethers

The ER and mitochondria are essential and highly connected intracellular organelles. The distance between the ER and OMM is approximately 10–25 nm, but this is highly variable as it can range from 10 to 100 nm (Csordas et al., 2006). This variable distance may result from the highly dynamic structure of these organelles under different conditions, such as decreased distance in response to ER stress (Bravo et al., 2011), hypoxia (Wu et al., 2016), and starvation (Sood et al., 2014) while an increased distance is shown with high glucose levels (Theurey et al., 2016). The ER interfaces with the mitochondria physically at mitochondria-ER contact sites, which are functionally defined as MAM. Diverse MAM morphologies have been observed using electron microscopy, such as ER tubules being tangential to mitochondria, ER tubules surrounding the mitochondria, and a portion of the ER tubules wrapping around approximately 50% of the mitochondrial circumference. All these morphologies can exist in the same cell (Lan et al., 2019).

**FIGURE 1:**
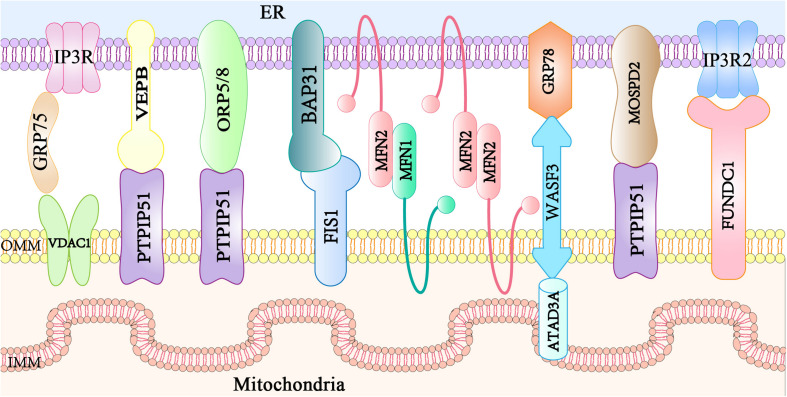
Main mitochondria–ER tethering complexes. Mitochondria are bridged to the ER by several protein complexes. The ER protein VAPB, ORP5/8, or MOSPD2 interacts with the mitochondrial PTPIP51. ER resident IP3R is anchored to OMM-localized protein VDAC via the cytosolic chaperone GRP75. The ER-localized MFN2 interacts with either MFN1 or MFN2 in the mitochondria. The ER protein BAP31 interacts with the mitochondrial FIS1. The ER chaperone GRP78, the cytosolic protein WASF3, and the IMM-localized ATAD3A form a tethering complex. The IP3R2 located on the ER partners with the mitochondrial protein FUNDC1.VAPB, vesicle-associated membrane protein-associated protein B; ORP, oxysterol-binding protein-related protein; MOSPD2, motile sperm domain-containing protein 2; PTPIP51, tyrosine phosphatase interacting protein 51; IP3R, inositol 1,4,5 trisphosphate receptor; VDAC, voltage-dependent anion channel; GRP75, glucose regulated protein 75; MFN, mitofusin; BAP31, B cell receptor-associated protein 31; FIS1, mitochondrial fission 1; GRP78, glucose regulated protein 78; WASF3, Wiskott-Aldrich syndrome protein family member 3; ATAD3A, ATPase family AAA domain containing 3A; FUNDC1, FUN14 domain-containing protein 1; ER, endoplasmic reticulum; MAM, mitochondria associated membranes; OMM, outer mitochondrial membrane; IMM, inner mitochondrial membrane.

Mitochondria-associated endoplasmic reticulum membranes are not directly involved in the membrane fusion with organelles, but they are composed of specific proteins located at the ER membrane, MAMs, and mitochondrial membrane. MAM protein complexes function as tethers to link ER and mitochondria together, and some regulatory proteins interact with and modulate these tethers. At least eight protein complexes have been identified to govern the ER-mitochondria bridging at MAM sites (Figure 1). The IP3R1-GRP75-VDAC1 complex consists of inositol 1,4,5-triphosphate receptors (IP3R1s) in the ER membrane and a VDAC1 at the OMM. Additionally, the 75-kDa glucose-regulated protein (GRP75) links IP3Rs and VDAC1 to maintain the conformational stability of IP3Rs that participate in Ca^2+^ transport from the ER to the mitochondria (Szabadkai et al., 2006). The VAPB-PTPIP51 complex (De Vos et al., 2012) is composed of ER resident VAPB involved in vesicle trafficking and the unfolded protein response (Kanekura et al., 2006), and mitochondrial protein tyrosine phosphatase interacting protein 51 (PTPIP51) that modulates cellular development and tumorigenesis (Yu et al., 2008). PTPIP51 also interacts with oxysterol-binding protein-related protein 5/8 (ORP5/8) localized at the ER membrane facing the cytosol, which is responsible for the phosphatidylserine (PS) transport to the mitochondria (Galmes et al., 2016). BAP31-FIS1 complex (Iwasawa et al., 2011) is formed from ER-localized BAP31 and mitochondrial fission 1 (FIS1), which participate in mitochondrial fission and apoptosis signaling (Yoon et al., 2003; Iwasawa et al., 2011). Mitochondria-shaping mitofusin 2 (MFN2) is localized both in the ER and mitochondria (de Brito and Scorrano, 2008), and homo- or hetero-typic interactions with mitochondrial mitofusin 1 (MFN1) mediate MAM contacts, mitochondrial Ca^2+^ uptake, or autophagosome formation (Naon et al., 2016; McLelland et al., 2018). The WASF3 in the cytoplasm interacts with the IMM protein ATPase family AAA domain-containing 3A (ATAD3A) by penetrating the OMM and binding to ATAD3A at its N-terminal region. This forms a complex with the ER protein, GRP78, constituting a new mitochondria-ER tethering complex, GRP78-WASF-ATAD3A, which promotes cell invasion in breast and colon cancer (Teng et al., 2016). The OMM-localized FUN14 domain-containing protein 1 (FUNDC1) directly binds to IP3R2 to form a bridge between the ER and the mitochondria, favoring Ca^2+^ flux to the mitochondria by enhancing the mitochondria-ER connection in cardiomyocytes (Wu et al., 2017). The loss of FUNDC1 promotes IP3R2 ubiquitination and degradation and decreases the levels of the MAM-maintenance protein phosphofurin acidic cluster sorting protein 2 (PACS2) (Wu et al., 2017). More recently, the ER-anchored motile sperm domain-containing protein 2 (MOSPD2) has been proposed as a tethering protein that interacts with PTPIP51 and functions in both intracellular exchange and communication (Di Mattia et al., 2018). Additional tethers or spacers will be identified in the future, allowing a more comprehensive understanding of the proteins involved in maintaining mitochondria-ER contacts. Thus, further studies are required to examine if the ablation of a single protein modulates the activity or localization of proteins involved in a different tether and to understand if these structures cooperate.

## Functions Linked to the MAMs

Mitochondria and ER bridging at the MAM regulates a variety of cellular functions, such as Ca^2+^ signaling, lipid trafficking, ROS production regulation, mitochondrial dynamics, and cell apoptosis. These functions are essential for controlling the physiological function of organelles and their coordination with each other as well as homeostasis in cellular metabolism. Dysfunctional mitochondrial-ER connections are responsible for the pathogenesis of many diseases.

## MAM in Calcium (Ca^2+^) Transfer

Ca^2+^ acts as an secondary intracellular messenger for signal transmission in multiple cellular processes (McMahon and Jackson, 2018), and the dysregulation of Ca^2+^ fluxes is involved in several human disorders (Giorgi et al., 2018). Basal Ca^2+^ oscillations in the mitochondria are pivotal for bioenergy generation and cell survival. This is because several mitochondrial enzymes involved in ATP synthesis via the tricarboxylic acid (TCA) cycle are regulated by Ca^2+^, such as α-ketoglutarate dehydrogenase, isocitrate dehydrogenase, pyruvate dehydrogenase, and ATP synthase (Pallafacchina et al., 2018). However, prolonged or excessive accumulation of Ca^2+^ in the mitochondria can lead to the opening of the mitochondrial permeability transition pore (mPTP) and subsequent release of pro-apoptosis factors (Rizzuto et al., 2012). The ER lumen serves as a storehouse for Ca^2+^, which is incorporated into the ER from the cytoplasm by sarcoplasmic/endoplasmic reticulum Ca^2+^-ATPase (SERCA). MAM acts as a bridge for Ca^2+^ transfer between the ER and mitochondria to balance the mitochondrial Ca^2+^ concentration level. There is an inverse relationship between mitochondrial Ca^2+^ uptake and MAM parameters, such as MAM distance, coupling expansion, and coupling number (Csordas et al., 2006). When the distance between MAM is shortened, the efficiency of Ca^2+^ transport will increase, which can easily lead to mitochondrial Ca^2+^ overload. As the distance increases, the efficiency of the Ca^2+^ transport decreases. However, when the distance between ER and mitochondria is less than 7 nm, the transport efficiency of Ca^2+^ will be reduced significantly because of the insufficient space for IP3R, suggesting that an optimal MAM distance is crucial to maintain Ca^2+^ transfer from the ER to the mitochondria (Csordás et al., 2010). Additionally, an increase in the MAM amount induced by obesity also results in mitochondrial Ca^2+^ overload (Arruda et al., 2014).

**FIGURE 2:**
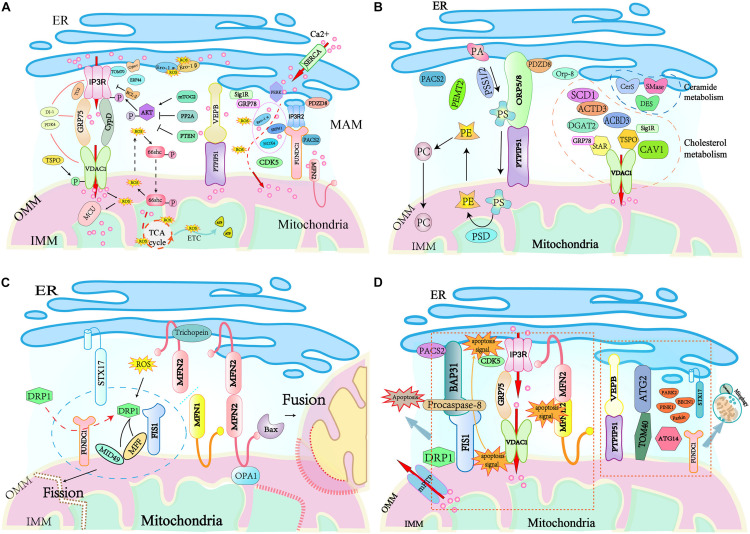
Key cellular functions handled at mitochondria–ER contact sites. **(A)** Ca^2+^ signaling and redox crosstalk occur in MAM. Ca^2+^ flux from ER to mitochondria through MAM tethering complex IP3R/VADC or PTPIP51/VERB and accumulates in the mitochondrial matrix by MCU transportation. The high amount of ROS accumulated at the MAM generates redox nanodomains. Several MAM resident proteins regulate Ca^2+^ signaling and ROS generation. **(B)** MAMs are hubs for lipid trafficking between the ER and mitochondria. MAM residing enzymes including PSS1, PSS2, PSD, PEMT2, cytochrome P450, SMase, CerS, and DES are responsible for the exchange of phospholipids, cholesterol, and ceramides between these two organelles. Mitochondria take PS from the ER, which is supplied with PE by mitochondria. **(C)** Mitochondrial fission and fusion are regulated at the interface between the ER and mitochondria. During mitochondrial fission, DRP1 recruited by MiD49/51 and MFF to the mitochondrial surface interacts with the ER-localized STX17. The ER-mitochondria contact is required for mitochondrial pre-constriction. DRP1 activity is modulated by redox signals. Mitochondrial fusion is promoted by trichoplein binding to ER-anchored MFN2, and this interaction favors the interaction of mitochondrion-bound MFN2 and OPA1 to initiate the fusion of mitochondrial membranes. **(D)** MAMs are involved in the initiation of apoptosis and the regulation of mitophagy/autophagy. MAM tethering complexes FIS1-BAP31, IP3R-GRP75-VDAC1, and MFN2-MFN1/2 bridges the mitochondria and the ER for apoptosis signaling. PACS2 modulates BAP31 function, CDK5 regulates mitochondrial Ca^2+^ homeostasis. Autophagosomes assembly arise from mitochondria-ER contacts, the ER-resident protein STX17 attracts ATG14 and the PI3K complex. Bax, Bcl-2 associated X protein; Bcl-2, B cell CLL/lymphoma 2; cyt. c, cytochrome c; DRP1, dynamin-related protein 1; HK2, hexokinase 2; MCL-1, myeloid cell leukemia sequence 1; MCU, mitochondrial calcium uniporter; mPTP, mitochondrial permeability transition pore; Orai1, ORAI calcium release-activated calcium modulator 1; PML, promyelocytic leukemia protein; PTEN, phosphatase and tensin homolog deleted on chromosome 10; SERCA, sarco/endoplasmatic reticulum Ca^2+^ ATPase; Sig1R, Sigma 1 receptor; STIM1, stromal interaction molecule 1; PA, phosphatidic acid; PS, phosphatidylserine; PE, phosphatidylethanolamine; PC, phosphatidylcholine; Preg, pregnolone; PSS, phosphatidylserine synthase, PSD, phosphatidylserine decarboxylase; PEMT2, phosphatidylethanolamine-*N*-methyltransferase 2.

Ca^2+^ is released from the ER to the mitochondria via the MAM tethering complexes IP3Rs-GRP75-VDAC1 or VAPB-PTPIP51 and finally accumulates in the mitochondrial matrix via the mitochondrial Ca^2+^ uniporter (MCU) complex (Figure 2A). Recent studies have identified numerous regulators of these tethering complexes at ER-mitochondria contacts. Mitochondria-related protein cytochrome c (Cytc) interacts with IP3Rs abolishing the Ca^2+^-mediated inhibition of IP3R-associated Ca^2+^ release, this promotes Ca^2+^ release (Boehning et al., 2003). While Bcl-2 (Ivanova et al., 2019), calreticulin (Camacho and Lechleiter, 1995), and ERp44 (Higo et al., 2005) inhibit the opening of IP3Rs and down-regulate IP3R-mediated Ca^2+^ flux. The OMM-localized 18 kDa translocator protein (TSPO) is demonstrated to inhibit mitochondrial Ca^2+^ uptake by promoting VDAC1 phosphorylation (Gatliff et al., 2017), while VDAC1 ablation in human glioblastoma U87 cells leads to reduced levels of TSPO (Shoshan-Barmatz et al., 2019). MFN2 depletion causes a decrease in mitochondrial Ca^2+^ uptake in the flexor digitorum brevis muscles (Ainbinder et al., 2015), but an increased mitochondrial Ca^2+^ overload in MEFs cells (Filadi et al., 2015). Cyclin-dependent kinase 5 (CDK5) was recently identified to be localized at MAM, and its absence in MAM enhances ER-mitochondria contacts, augmenting the Ca^2+^ uptake in the mitochondria (NavaneethaKrishnan et al., 2020). Cyclophilin D (CypD) is a partner of the IP3R1-GRP75-VDAC1 complex in the liver. It not only changes the MAM spatial structure but also interacts with and maintains the tethering complex (Tubbs et al., 2014). Pyruvate dehydrogenase kinase 4 (PDK4) directly interacts with and stabilizes the IP3R1-GRP75-VDAC1 complex at the MAM interface, and obesity-induced increases in PDK4 activity augment the MAM formation and Ca^2+^ flux to the mitochondria (Thoudam et al., 2019). The ER protein sigma 1 receptor (Sig1R) is also localized to MAM, and it forms a Ca^2+^-sensitive chaperone complex with GRP78 to ensure the normal Ca^2+^ transfer between the ER and mitochondria by stabilizing the conformation of IP3Rs (Parys and Vervliet, 2020). Mammalian TOR complex 2 (mTORC2) in the MAM phosphorylates and activates AKT in response to insulin stimuli (Yoon, 2017), and the active AKT phosphorylates IP3Rs and inhibits IP3R Ca^2+^ release (Bassot et al., 2019; Thoudam et al., 2019). In contrast, protein phosphatase 2A (PP2A) recruited by promyelocytic leukemia protein (PML) in the MAM inactivates AKT and facilitates IP3R-mediated Ca^2+^ release from the ER (Giorgi et al., 2010). PDZ domain-containing protein 8 (PDZD8) localizes at the ER fraction of the MAM, and the reduction of PDZD8 attenuates both mitochondria-ER contacts and Ca^2+^ flux into the mitochondria in HeLa cells (Hirabayashi et al., 2017). More recently, transglutaminase type 2 (TG2), DJ-1, and TOM 70 have been shown to regulate the Ca^2+^ flux by modulating the stability of the IP3R3-GRP75-VDAC1 complex. TG2 regulates the interaction between IP3R3 and GRP75 and the number of ER-mitochondria contacts by binding to GRP75 under conditions of cellular stress (D’Eletto et al., 2018). DJ-1 physically interacts with the IP3R3-GRP75-VDAC1 complex, and loss of DJ-1 disrupts the tethering complex and leads to disturbed Ca^2+^ efflux from the ER (Liu et al., 2019). TOM70 interacts with IP3R3, which promotes their functional recruitment close to the mitochondria, facilitating the transfer of Ca^2+^ to the mitochondria (Filadi et al., 2018). Furthermore, ER oxidoreductin-1 (Ero1) α (Anelli et al., 2012), NADPH oxidase 4 (NOX4) (Sun et al., 2011), and selenoprotein N1 (SEPN1) (Pitts and Hoffmann, 2018) are also involved in the regulation of Ca^2+^ transfer at MAMs. Given that they are important regulators of the ER redox status, it is proposed that the oxidation state of ER can also regulate the ER- mitochondria Ca^2+^ flux (Chernorudskiy and Zito, 2017).

## MAM Participates in Reactive Oxygen Species (ROS) Generation

Reactive oxygen species, an oxygen-containing reactive species including H_2_O_2_ and singlet oxygen, is formed as a result of the utilization of molecular oxygen under both normal metabolism and stress conditions (Sies and Jones, 2020). The role of ROS in cells can be beneficial or hazardous, and this is determined by its intercellular concentration. At normal levels, ROS functions as a “redox messenger” modulating many physiological events, including metabolism, hypoxia adaptation, autophagy, and immune cell activation. In contrast, excessive ROS compromises protein function and accelerates inflammation and cell death by inducing oxidative modification of cellular macromolecules (Stowe and Camara, 2009; Camara et al., 2010). Both the mitochondria and ER are sites of intracellular ROS generation (Holmström and Finkel, 2014), which occurs at the electron transport chain during the processes of oxidative metabolism and ATP synthesis in mitochondria (Murphy, 2009), and ROS is produced during the process of oxidative protein folding mediated by Ero1-α and -β in ER (Benham et al., 2013). Not surprisingly, the mitochondria-ER connection participates in ROS diffusion between organelles, promoting its harmful effects on the cell (Figure 2A). An example of this is the ER stress sensor PERK, which is enriched in MAM and acts as a modulator to ease Ca^2+^ influx into mitochondria during ER stress (van Vliet et al., 2016). The influx of Ca^2+^ into mitochondrial cristae evokes the production of a large amount of ROS, which further promotes Ca^2+^ flux to the mitochondrial matrix by oxidizing MCU (Feissner et al., 2009). Furthermore, uptake of Ca^2+^ to the matrix stimulates the translocation of ROS from mitochondrial cristae to MAM, resulting in the generation of redox nanodomains at the mitochondria-ER interface (Booth et al., 2016).

The increased ROS at the MAM not only enhances Ca^2+^ flux from the ER to the mitochondria by targeting ROS effectors that mediate the local Ca^2+^ homeostasis of the two organelles (Csordás and Hajnóczky, 2009), but it also modulates mitochondria-ER apposition through the MAPK-dependent control of mitochondrial mobility (Debattisti et al., 2017). Therefore, mitochondria-derived ROS might be an inducer that amplifies the Ca^2+^ release feedback signaling at the MAM interface. ROS has been shown to stimulate the oxidation of the ER Ca^2+^-channel ryanodine receptor (RyR), resulting in the enhancement of both Ca^2+^ leakage and ROS production (Andersson et al., 2011). Other MAM Ca^2+^-transporting proteins, such as cytoplasmic kinases and phosphatases are also ROS-dependent regulators (Giorgi et al., 2015). Additionally, many MAM structural proteins are directly involved in the mitochondria-ER redox crosstalk (Myhill et al., 2008; Li et al., 2009; Marino et al., 2015). One classic example is the feedback loop regulation between the activation of the 66-kDa Shc isoform (p66Shc) and mitochondrial ROS generation (Yang et al., 2014). The MAM-localized p66Shc is first phosphorylated at the Ser36 residue by oxidative stress insults and, in turn, moves into the mitochondria to induce the production of H_2_O_2_ (Bhat et al., 2015). It is well-known that the redox state at the MAM modulates the activity of the MAM-enriched protein Ero1-α (Fan and Simmen, 2019), which mediates the formation of H_2_O_2_ and, in turn, potentiates Ca^2+^ signaling at the MAM and the consequent oxidation of the IP3R (Anelli et al., 2012). More recently, it has been shown that aquaporin-11 at MAM is required for H_2_O_2_ flux through the ER, indicating the potential role of MAM in ER redox homeostasis (Bestetti et al., 2020). Altogether, these data indicate that MAM plays an essential role in ROS accumulation and signaling.

## MAM in the Synthesis and Trafficking of Lipids

Lipids are physiologically active substances that have various biological functions, including energy storage and signal transduction. Although mitochondrial phospholipids such as cardiolipin (CL), phosphatidylethanolamine (PE), and phosphatidylglycerol (PG) are synthesized solely by the mitochondria, most lipids such as phosphatidic acid (PA), phosphatidylinositol (PI), phosphatidylserine (PS), phosphatidylcholine (PC), and sterols are imported from the ER. The ER is the primary cellular “lipid factory” synthesizing the bulk of structural phospholipids, sterols, and storage lipids such as triacylglycerols and steryl esters.

The MAMs harbor several proteins or enzymes involved in lipid synthesis and trafficking, and these control relevant biological processes, including cell fate (Figure 2B). Firstly, some MAM-enriched enzymes are essential for the synthesis of lipids. These enzymes include the long-chain fatty acid-CoA ligase 4 (FACL4/ACS4) that activates long-chain fatty acids for the synthesis of complex lipids or acylated proteins (Lewin et al., 2001), stearoyl-CoA desaturase-1 (SCD1) that catalyzes the biosynthesis of monounsaturated fatty acids from saturated substrates (Man et al., 2006), DGAT2 that catalyzes the final step of triacylglycerol synthesis (Stone et al., 2009), and ACAT1/SOAT1 that converts intracellular free cholesterol into cholesteryl ester (Rusinol et al., 1994). The alteration of MAM-maintaining proteins has an impact on lipid anabolism. In ORP8-depleted mice, there is a marked elevation in HDL-cholesterol and phospholipids. Additionally, primary ORP8-deficient hepatocytes secret nascent HDL particles, suggesting an alteration in HDL biosynthesis (Beaslas et al., 2013). Human fibroblasts with deletions in the ATAD3 gene cluster display perturbed cholesterol and lipid metabolism (Desai et al., 2017). PACS2 depletion diminishes the levels of the fatty acid metabolism enzymes phosphatidylserine synthase (PSS) 1 and FACL4 in human skin melanoma cells (Simmen et al., 2005). Secondly, MAM is responsible for the functional transition of different lipid species and phospholipid exchange between the ER and mitochondria. A classic example is the biosynthesis of PS from PA in the ER by the PSS 1 and 2 (Stone and Vance, 2000). After translocation to the mitochondria via the MAMs lipid transfer tethering ORP5/8-PTPIP51, PS is converted into PE by phosphatidylserine decarboxylase (PSD). The newly synthesized PE is then transported out of the mitochondria, and is subsequently methylated by the MAM-enriched PEMT2 to generate PC. Moreover, MAM is also involved in cholesterol transfer. MAM-associated caveolin-1 (CAV1) plays a vital role in cholesterol efflux, and silencing CAV1 in a hepatic MAM will lead to aberrant intracellular free cholesterol accumulation, as well as the reduced physical extension and integrity of MAM (Sala-Vila et al., 2016). Similarly, MAM-enriched ATAD3 and Acyl-CoA-binding domain-containing 3 (ACBD3) are believed to participate in the regulation of cholesterol transport and steroidogenesis (Liu et al., 2006; Issop et al., 2015). Under a stress response or hormonal stimulation, MAM-associated StAR and TSPO interact with VDAC1 to restrict cholesterol transport, resulting in cholesterol accumulation in mitochondria and the subsequent mitochondrial steroidogenesis (Bose et al., 2008). More recent evidence suggests that GRP78 regulates StAR folding to ensure the high activity of StAR at the MAM, thereby being an acute regulator of steroidogenesis (Prasad et al., 2017). Finally, MAM-residing enzymes, such as sphingomyelin phosphodiesterase (SMase), ceramide synthase (CerS), and dihydroceramide desaturase (DES) are necessary for ceramide biosynthesis. Given that mitochondrial ceramide is a critical player in the induction of apoptosis, MAM might act as a major ceramide reservoir or a critical barrier to alleviate mitochondrial ceramide overload (Mignard et al., 2020). However, all the available evidence for the role of MAM-associated proteins in lipid trafficking is derived from cultured cells, and further studies are required to prove their functions *in vivo*.

## MAM Is Crucial for Mitochondrial Dynamics and Function

The mitochondria are highly dynamic endosymbiotic organelles with an OMM, IMM, and circular mitochondrial DNA (mtDNA) (Nunnari and Suomalainen, 2012). Mitochondria have variable morphologies that are determined by fusion and fission, and they move along the cytoskeleton to become fragmented or tubular mitochondrial networks in response to cellular energy demands and apoptotic stimuli (Nunnari and Suomalainen, 2012; Wai and Langer, 2016). Mitochondria-ER contacts are crucial for mitochondrial fusion and fission (Figure 2C). Mitochondrial fusion involves fusing the OMM and IMM of one mitochondrion to another, which relies on the dynamin-related GTPases optic atrophy 1 (OPA1) in IMM and MFN1 and MFN2 in the OMM (Chan, 2006; Hailey et al., 2010). MFN2 acts as a mitochondria-ER tethering complex, which is inhibited by the MAM protein trichoplein when it is bound to MFN2 at the ER, favoring mitochondrial fusion (Cerqua et al., 2010). MFN2 also interacts with pro-apoptotic Bcl-2 protein Bax to promote mitochondrial fusion in healthy cells, where the soluble, monomeric Bax regulates MFN2 complex assembly at sites of mitochondrial fusion (Karbowski et al., 2006; Hoppins et al., 2011). Furthermore, the OMM-localized FUNDC1 can interact with the IMM protein OPA1 to promote mitochondrial fusion under normal conditions (Chen et al., 2016).

Mitochondrial fission is mainly driven by the dynamin-related protein 1 (DRP1, or DNM1 in yeast), which is mostly cytoplasmic (Smirnova et al., 2001). DRP1 is recruited to the mitochondria by FUNDC1 under hypoxic conditions and interacts with its adaptor proteins, such as FIS1, MFF, MiD49, and MiD51 to promote mitochondrial fission, resulting in mitochondrial fragmentation (Wu et al., 2016). Mitochondria-bound DRP1 can also interact with the ER protein syntaxin 17 (STX17) at mitochondria-ER contact sites to support fission (Arasaki et al., 2015). The activity of DRP1 is regulated by phosphorylation modification, and DRP1 Ser637 is the most studied phosphorylation site mediated by protein kinase A (PKA) (Chang and Blackstone, 2007) or Ca^2+^/calmodulin-dependent protein kinase Iα (CaMKIα) (Han et al., 2008) depending on the cellular context in which it occurs. The phosphorylation of Ser637 by PKA inhibits fission activity through a reduction in GTPase activity with or without inhibition of DRP1 translocation to mitochondria. In contrast, phosphorylation by CaMKIα in response to increased intracellular Ca^2+^ concentrations increases fission activity, perhaps through an increase in DRP1-binding affinity for FIS1, with or without stimulation of the mitochondrial translocation of DRP1. A recent study found that DRP1 Ser637 dephosphorylation mediated by calcineurin promotes DRP1 recruitment to the mitochondria (Cereghetti et al., 2008). DRP1 activity is also modulated by redox signals, and elevated ROS in the mitochondria results in DRP1 oxidation, and increased mitochondrial division that favors ROS accumulation in the mitochondria (NavaneethaKrishnan et al., 2020). Given the fundamental role of MAM in cellular Ca^2+^ signaling and ROS generation, it is not surprising that MAM plays a crucial role in the regulation of DRP1 activity and mitochondrial fission. Before DRP1 recruitment, mitochondrial fission at ER-mitochondria contacts has also been observed (Friedman et al., 2011). Thus, the actin filaments at ER-mitochondria contacts may drive primary mitochondrial constriction, which allows DRP1-driven secondary constriction that induces mitochondrial fission (Korobova et al., 2013). Furthermore, ER-mitochondria contact sites are the positions that mediate mtDNA synthesis and the distribution of the nascent mtDNA to the daughter mitochondria after fission (Lewis et al., 2016). However, it is unclear how mitochondrial division is coordinated with the mtDNA replication.

## MAM Modulates ER Stress

The ER is also an important organelle for protein synthesis, folding, post-translation modification, and secretion in eukaryotic cells, in addition to being a storehouse for Ca^2+^ (Liu et al., 2017). Cells have evolved a complete set of mechanisms to regulate protein folding and modification in the inner cellular network. When misfolded proteins accumulate in the lumen, ER homeostasis is disturbed, and ER stress occurs. Cells respond to ER stress by initiating a series of signaling pathways mainly mediated by the ER-localized sensor protein kinase IRE1α, PERK, and ATF6, which are typically kept inactive by GRP78. The activity of these proteins leads to an ER-specific unfolded protein response (UPR) (Hetz and Papa, 2018).

Mitochondria-associated endoplasmic reticulum membranes proteins modulate ER stress signaling, and the classical tethering protein MFN2 can bind to PERK and repress its activity (Muñoz et al., 2013). MFN2 depletion in MEF cells causes PERK activation and an enhanced PERK-EiF2α-ATF4-CHOP pathway, PERK silencing in these cells can restore the mitochondrial Ca^2+^ contents and improve mitochondrial morphology (Muñoz et al., 2013). The specific ablation of Mfn2 in mouse cardiac myocytes upregulates the expression of ER stress markers BiP, GRP94, and ATF4 (Xin et al., 2019). The tethering protein VAPB interacts with ATF6 directly to repress the UPR (Muñoz et al., 2013), the forced overexpression of VAPB both in HEK293 and NSC34 cells attenuates ATF6 transcription. Additionally, the loss of other MAM proteins, such as PACS2 (Simmen et al., 2005), Sig1R (Hayashi and Su, 2007), Mfn2 (de Brito and Scorrano, 2009), or CypD (Rieusset et al., 2016), also induces ER stress by disrupting the ER-mitochondria communication. Moreover, the alteration of ER stress proteins also modulates mitochondria-ER contacts and the associated biological processes (Gutierrez and Simmen, 2018). An initial ER stress promotes the mitochondria-ER connection, which enhances mitochondrial ATP production and Ca^2+^ uptake, contributing favorably to the cellular adaptation to ER stress (Bravo et al., 2011). Chronic UPR signaling triggers a signaling cascade in the MAM that ultimately results in apoptosis (Hetz and Papa, 2018). PERK is uniquely enriched at MAMs and is involved in maintaining the mitochondria-ER contacts and enhancing the ROS-induced mitochondrial apoptosis (Verfaillie et al., 2012). IRE1α enriched at the MAM has dual functions in deciding cell fate-promoting cell survival by splicing the xbp1 mRNA or inducing cell death by mitochondrial Ca^2+^ overload (Carreras-Sureda et al., 2019). A recent study reported that IRE1α ubiquitylation by ubiquitin ligase at MAM could hinder ER stress-induced apoptosis (Takeda et al., 2019).

## MAM Regulates Apoptosis

Cells initiate apoptotic pathways when they cannot adequately handle specific stress insults. MAMs harbor diverse regulators and signaling pathways for apoptosis (Figure 2D). The MAM tethering FIS1-BAP31 complex directly participates in the apoptosis process by transmitting apoptotic signals from the mitochondria to the ER (Iwasawa et al., 2011). The OMM protein FIS1 enhances the recruitment of DRP1 to mitochondrial division sites (Stojanovski et al., 2004), while the ER chaperone BAP31 regulates the degradation of the misfolded protein and the apoptotic pathway (Nguyen et al., 2000). The mitochondrial apoptosis signal to the ER is conveyed by the FIS1 physically interacting with BAP31 and inducing the caspase-dependent cleavage of BAP31. This process activates procaspase-8 that is recruited to MAM by the FIS1-BAP31 tethering complex. Therefore, the FIS1-BAP31 complex bridges the mitochondria and the ER for apoptosis signaling (Iwasawa et al., 2011). The tether IP3R-GRP75-VDAC1 complex also facilitates the apoptosis pathway. Apoptosis induced by either the extrinsic or intrinsic apoptotic pathway was reduced effectively by silencing IP3R (Mendes et al., 2005). Similarly, downregulation of the expression of VDAC1 selectively rescues the apoptosis pathway induced by the low-amplitude apoptotic Ca^2+^ signal transduction (De Stefani et al., 2012). Moreover, the integrity and stability of the MAM is also crucial for cell survival. Mitochondrial fragmentation and dissociation from the ER triggers apoptosis, and mitochondrial division occurs with apoptotic cell death (Youle and Karbowski, 2005). Conversely, mitochondrial fusion protects cells from apoptosis, inhibition of MFN2-mediated fusion renders cells more sensitive to apoptotic stimulation (Sugioka et al., 2004; Neuspiel et al., 2005). Hence, the MAM tether, MFN2-MFN1/2 that mediates mitochondrial fusion, can regulate the apoptotic pathway via the regulation of MAM stability (Sugioka et al., 2004). PACS2 modulates the mitochondria-ER contacts, and the absence of PACS2 impairs the function of the MAM tethering protein BAP31, leading to BAP31-dependent mitochondrial fragmentation and disassociation from the ER (Simmen et al., 2005). Recent evidence shows that CDK5 in MAMs regulates mitochondrial Ca^2+^ homeostasis, where the loss of CDK5 in the MAM causes an increase in Ca^2+^-induced mPTP opening and subsequent cell apoptosis (NavaneethaKrishnan et al., 2020).

## MAM Regulates Autophagy

Autophagy is a self-degradative process that removes the misfolded and aggregated proteins or damaged components from the cytoplasm. When autophagy occurs, autophagosomes engulf damaged or unnecessary components and fuse with lysosomes to degrade their contents. MAM itself is required for autophagosomes formation and probably facilitates autophagosomal membrane formation (Hailey et al., 2010; Hamasaki et al., 2013) (Figure 2D). Autophagy-related 2 (ATG2) is a rod-shaped protein that tethers phagophores to the ER, accumulates at MAMs, and interacts with the mitochondrial translocase TOM70 to promote autophagic membranes growth during nutrient starvation (Tang et al., 2019). Depletion of TOM70 impairs mitochondrial respiration, weakens cell bioenergetics, inhibits cell proliferation, and initiates autophagy (Filadi et al., 2018). The MAM tethering proteins play crucial roles in autophagosome formation. Overexpression of VAPB or PTPIP51 reduces autophagosome formation by enhancing ER-mitochondria contacts. Conversely, loss of mitochondria-ER contacts by silencing VAPB or PTPIP51 induces autophagosome formation (Gomez-Suaga et al., 2017). Under starvation conditions, ATG14 recruited to the MAM by STX17 initiates autophagosome formation and ablation of MAM-associated MFN2, or PACS2 hinders STX17-mediated ATG14 recruitment and autophagosome biogenesis (Hamasaki et al., 2013). Mitophagy is a form of autophagy, which ensures that mitochondria with normal quality and metabolism are maintained, and damaged mitochondria are removed, thus promoting cell survival (Palikaras et al., 2018). When cells encounter a hypoxic stimuli, critical enzymes of mitophagy such as PINK1, BECN1, PARK2, Parkin, and FUNDC1 increasingly accumulate at the MAM to trigger the formation of autophagosomes favoring removal of the damaged mitochondria (Chen et al., 2016; Gelmetti et al., 2017).

## MAM Dysfunction in T2DM

Increasing evidence shows that aberrant Ca^2+^ signaling, ROS generation, ER stress, and alteration of autophagy are implicated in the insulin resistance seen in peripheral tissues (Rieusset, 2015; Pinti et al., 2019), and these are crucial to β cell function (Wang et al., 2016). Considering important functions played by MAM in Ca^2+^ homeostasis, cellular ROS production, and ER stress, the dysfunction of MAM could be involved in the development of T2DM. Therefore, MAM activity is increasingly becoming a new potential mechanism to explain T2DM, and maintenance of the proper function of MAM might be a novel therapeutic strategy to address T2DM.

## MAM Participates in Insulin Signaling

Normally, insulin is released from the β cells of the pancreatic islets. Insulin increases glucose uptake in muscle and adipose tissues while decreasing hepatic glucose production (Zhang et al., 2018), as well as mediating cell growth and differentiation, stimulating lipogenesis, enhancing glycogen and protein synthesis, and inhibiting lipolysis, glycogenolysis, and protein breakdown (Saltiel and Kahn, 2001). The MAMs play a vital role in insulin signaling, and this is because several insulin signaling proteins such as AKT kinase, mTORC2, PP2A, and PTEN are detected at the MAM contacts (Figure 2A). The AKT kinase plays a central role in insulin signaling, as it is activated via phosphorylation after insulin stimulation at the MAM interface. The MAM-localized kinase, mTORC2, whose level in the MAM is increased in response to insulin stimulation or growth factors, mediates phosphorylation-activating AKT (Betz et al., 2013). Activated AKT subsequently phosphorylates IP3R and inhibits the IP3R-mediated release of Ca^2+^ (Giorgi et al., 2010), which is required for insulin signaling in both cardiomyocytes (Gutierrez et al., 2014) and skeletal muscle cells (Navarro-Marquez et al., 2018). In addition, AKT also phosphorylates MAM resident proteins PACS2 (Aslan et al., 2009) and hexokinase 2 (HK2) (Miyamoto et al., 2008) to maintain MAM integrity, which is necessary for insulin signaling (Tubbs et al., 2014). MAM disruption in hepatocytes leads to compromised insulin signaling, while reinforcement of the integrity of MAM enhances insulin action. The activity of AKT and its associated inhibitory action on Ca^2+^ release is regulated by the MAM-localized PP2A and the tumor suppressor PTEN, whose phosphatase activity catalyzes the dephosphorylation of AKT and IP3R, respectively, thus counteracting AKT-mediated phosphorylation, restoring Ca^2+^ translocation from the ER to the mitochondria, and sensitizing cells to apoptosis (Bononi et al., 2013). Recently, nitric oxide (NO) has been suggested to increase the MAM integrity by activating the soluble guanylate cyclase and protein kinase G pathway, while simultaneously improving hepatic insulin sensitivity (Bassot et al., 2019). Furthermore, a recent study demonstrated that obesity-induced increases in PDK4 activity augments MAM stability and suppresses skeletal muscle insulin signaling (Thoudam et al., 2019). Similarly, disruption of insulin signaling by silencing critical proteins involved in the process, such as AKT or mTORC2, also inevitably impairs the MAM integrity and mitochondrial function (Betz et al., 2013), suggesting a reciprocal relationship between insulin signaling and MAM integrity. Although there is no doubt that MAM participates in insulin signaling, the underlying mechanism is unclear.

## MAM Is Altered in the Insulin Resistance of Peripheral Tissues

**FIGURE 3:**
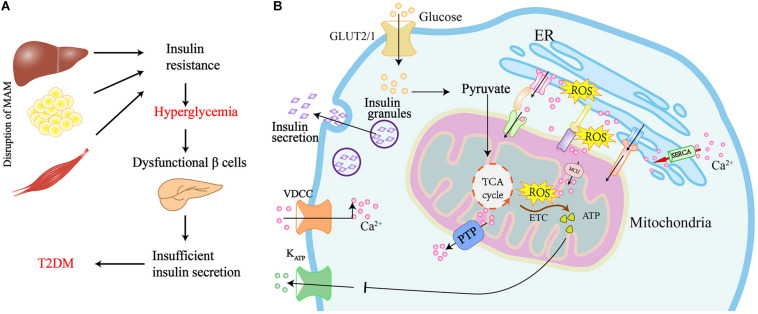
Graphical summary of the MAM implicated in the insulin resistance of peripheral tissues and insulin secretion in pancreatic β cells. **(A)** Disruption of MAM leads to insulin resistance and the development of T2DM. **(B)** Scheme of MAM involved in the regulation of insulin secretion in pancreatic β cells. Glucose uptake via glucose transporter-2 (GLUT2) boosts mitochondrial ATP production and ROS generation by mitochondrial electron transport chain (ETC). Elevated ATP leads to the closure of ATP sensitive K^+^ channels (KATP) and the activation of voltage-dependent Ca^2+^ channels (VDCC), which ultimately results in the exocytosis of insulin-containing granules. Ca^2+^ flux into mitochondria via the MAM tethering complex increases ROS generation, which further promotes Ca^2 +^ flux to the mitochondrial matrix by oxidizing the mitochondrial Ca^2+^ uniporter (MCU). The translocation of ROS from cristae to MAM results in the generation of redox nanodomains at the mitochondria-ER interface.

The presence of T2DM is confirmed by high glucose levels during both the fasting and post-prandial state, and this is because of insulin resistance in the peripheral tissues (Kahn et al., 2006; Chen et al., 2019). Given that MAM harbors several proteins responsible for the insulin signaling, and its integrity is also required for insulin action, it is not surprising that MAM alternations occur in the presence of insulin resistance and T2DM (Figure 3A).

The integrity of MAM is altered in both palmitate-induced insulin-resistant HuH7 cells and the livers of obese and diabetic mice. Liver-specific knockout of MFN2 or the inactivation of mTORC2 impairs MAM integrity, inducing multiple disordered metabolic bioprocesses, including insulin resistance and glucose tolerance (Sebastián et al., 2012). Similarly, genetic deletion or pharmacological inhibition of CypD interrupts MAM integrity and induces hepatic insulin resistance in mice, while CypD overexpression in mice primary hepatocytes increases mitochondria-ER contacts and improves insulin action (Rieusset et al., 2016). Furthermore, antidiabetic drugs, such as rosiglitazone or metformin can restore the MAM contacts and alleviate insulin resistance in diabetic mice (Tubbs et al., 2014). Additionally, MAM disruption is also an essential subcellular alteration that promotes muscle insulin resistance in mice and humans. MAMs with aberrant structure and function are seen in both the skeletal muscle of diabetic mouse models and insulin-resistant human myotubes induced by palmitate treatment (Tubbs et al., 2018). Moreover, the number of mitochondria-ER contacts is decreased in myotubes derived from obese and diabetic patients (Tubbs et al., 2018). Reinforcement of MAM contacts by the forced overexpression of Grp75 or Mfn2 reverses the impaired effects of palmitate on MAM proteins and insulin signaling in skeletal cells (Tubbs et al., 2018). Except for the liver and skeletal muscle, insulin resistance in adipose tissue also plays a crucial role in T2DM. Elevated release of FFAs from insulin-resistant adipose tissue inhibits insulin secretion of the β cell, reduces glucose uptake in muscle, and increases glucose production from the liver (Saltiel and Kahn, 2001). Despite this, few studies have directly demonstrated the relevance of MAMs and adipose tissue in the metabolism and insulin sensitivity seen in T2DM.

In contrast to the disruption of MAM contributing to the insulin resistance, recent evidence has shown that the enhancement of the MAM formation also promotes insulin resistance and the development of diabetes with the dysfunctional organelles seen in obesity (Arruda et al., 2014; Thoudam et al., 2019). Insulin-resistant and obese (ob/ob) mice and high-fat diet (HFD) induced obese mice exhibit a significant increase of MAM formation with increased MFN2, IP3R1, and PACS2 in MAM fractions. This induces mitochondrial Ca^2+^ accumulation and oxidative stress augmentation in the liver, leading to mitochondrial dysfunction and insulin resistance (Arruda et al., 2014). Conversely, the interference of Ca^2+^ flux to the mitochondria by downregulating the MAM proteins PACS2 or IP3R1 improves mitochondrial oxidative capacity and insulin sensitivity (Arruda et al., 2014). Moreover, PACS2 knockout mice exhibit an increase in hepatic FGF21 expression and resistance to diet-induced obesity (Krzysiak et al., 2018). FUNDC1 is also a crucial MAM tethering protein, FUNDC1-ablated mice treated with a HFD diet exhibit improved glucose handling, insulin sensitivity, and less adiposity (Wu et al., 2019). Furthermore, FUNDC1- and PACS2-deficient mice show highly activated FGF21 that systemically modulates energy homeostasis and insulin sensitivity (Jimenez et al., 2018), and this might be another explanation of the improved insulin signaling observed in these mice. Similarly, the increased PDK4 activity in ob/ob mice and HFD-fed mice augments MAM formation by interacting with and stabilizing the IP3R1-GRP75-VDAC1 tethering complex and accelerating insulin resistance in skeletal muscle. In contrast, deletion or inhibition of PDK4 attenuates insulin resistance by reducing the MAM contents and MAM-induced mitochondrial Ca^2+^ overload (Thoudam et al., 2019).

Together, an increasing amount of evidence indicates that mitochondria-ER contacts are altered in various metabolic tissues derived from patients with obesity, T2DM, and insulin resistance. However, there is considerable discrepancy in the available data. The divergence between studies may result from the different mouse models and their different metabolic status, differences in the methodology used, or the multiple roles of MAM in insulin resistance. Moreover, considering that MAM proteins are not specific for ER-mitochondria connections, non-specific effects may occur when modulating their expression. Accordingly, further studies should focus on the dynamic change of the MAM and demonstrate links between mitochondrial dysfunction and insulin resistance. Furthermore, it is unclear if the alteration of mitochondria-ER contacts can modulate insulin sensitivity directly, or if on the contrary, insulin resistance affects these organelle contacts. Further investigations are required to reveal if modulating the expression, activity, or localization of proteins in the MAM interface has an impact on insulin action and if these strategies can be applied to the treatment of insulin resistance and T2DM.

## MAM Modulates the Secretory Function and Mass of the β Cell

Under physiological conditions, pancreatic β cells modulate insulin secretion in response to elevated levels of blood glucose to maintain glucose homeostasis. As shown in Figure 3B, blood glucose is taken up into β cells through glucose transporter-2 (GLUT2) and metabolized to pyruvate by glycolysis. Pyruvate enters the mitochondria to promote ATP production using the TCA cycle and, subsequently, the mitochondrial electron transport chain (ETC). The increased ATP inhibits ATP-sensitive K + channels, leading to depolarization of the plasma membrane and activation of voltage-dependent Ca^2+^ channels. This ultimately results in an influx of Ca^2+^ in the cell, thereby triggering the release of secretory granules containing insulin. Having seen how insulin is released from β cells, it is conceivable that the mitochondria play a central role in β cell function (Las et al., 2020). Insulin is synthesized and folded at the ER of pancreatic β cells. Dysregulation of ER will stimulate ER stress and the subsequent activation of UPR, which has an impact on whether the β cell survives or undergoes apoptosis (Ghosh et al., 2019).

Given that MAM plays a critical role in the mitochondria-ER communication and the ER-mitochondria contact sites are the major determinants of intracellular Ca^2+^ homeostasis, proper Ca^2+^ distribution is crucial for insulin production and secretion by pancreatic β cells. Therefore, the dysfunctional MAM in pancreatic β cells is also considered a major contributor to T2DM (Figure 3B). Indeed, recent evidence has shown that interactions between the ER and mitochondria are decreased in β cells from patients with T2DM (Thivolet et al., 2017). Additionally, palmitate-induced ER stress in Min6 β cells leads to a reduction of both mitochondria-ER contacts and insulin secretion (Thivolet et al., 2017). Furthermore, the alteration of MAM tethering proteins also affects the function and mass of β cells. IP3R1-depleted mice demonstrate impaired ER Ca^2+^ homeostasis, ER stress, compromised glucose tolerance, and reduced β cell mass (Takeda et al., 2016). In contrast, depleting VDAC1 in pancreatic β cells leads to enhanced ATP generation and the subsequent plasma membrane depolarization with increased cytosolic Ca^2+^ and insulin secretion, which protects β cells against high glucose levels and maintains the reductive capacity of cells (Zhang et al., 2019). INS1 β cells also upregulate the expression of VDAC1 in response to the elevated glucose levels in the culture medium. In line with these results, silencing VDAC1 in pancreatic islets from diabetic (db/db) mice results in increased ATP production and glucose-stimulated insulin exocytosis in response to high glucose levels (Zhang et al., 2019). These findings prove the importance of MAM in β cells during the process of T2DM, but the underlying regulatory mechanisms of MAM are still unclear and require further investigation. The emerging role of the ER-mitochondria contacts in the dysfunction of pancreatic islets and peripheral tissues, suggests that maintaining the proper function of MAM might be a novel new therapeutic strategy for T2DM.

## Perspective

The mitochondria-ER contacts are gaining importance because of their physiological implications, and their role as novel and efficient therapeutic targets for T2DM. However, although numerous proteins and their regulators are localized in MAM, no proteins are exclusively expressed at the MAM interface. The full composition of proteins and tethering complexes involved in the control of the elegant structure and function of MAM still needs to be discovered, and this will demonstrate new molecular mechanisms underlying the regulation of such a conserved connection between the cellular organelles.

Overall, the findings discussed in this review indicate that the disruption of MAM contributes to insulin resistance, dysfunctional pancreatic β cells, and the consequent T2DM. MAM harbors many proteins involved in Ca^2+^ signaling, redox homeostasis, mitochondrial metabolism, ER stress, and autophagy which participate directly or indirectly in the control of glucose homeostasis. In this context, targeting the MAM could be employed to find more efficient pharmacological approaches for the treatment of T2DM. Currently, although drugs alleviating ER or mitochondrial stress have been shown to enhance insulin action and glucose homeostasis, in both animal models and patients, there are no strategies targeting MAM for the cure of insulin resistance of peripheral tissues or insufficient insulin secretion. Given the dynamic properties of MAM and the fact that the proteins are not specific to the MAM interface, future studies should be focused on the identification of putative MAM targets to develop novel drugs for the treatment of specific clinical conditions. Overall, we believe that modulating the insulin action precisely in the desired cells by targeting the cell type-specific MAM, would be an attractive strategy that does not affect the homeostasis of other cells.

## Author Contributions

All authors listed have made a substantial, direct and intellectual contribution to the work, and approved it for publication.

## Conflict of Interest

The authors declare that the research was conducted in the absence of any commercial or financial relationships that could be construed as a potential conflict of interest.

## Abbreviations

ACAT1: acyl-coenzyme-A-cholesterol acyltransferase 1
ACBD3: acyl-CoA binding domain containing 3
ATAD3A: ATPase family AAA domain containing 3A
ATF6/4: activating transcription factor 6/4
ATP: adenosine triphosphate
BAP31: B-cell receptor-associated protein 31
Ca^2+^: calcium
CAV1: caveolin-1
CDK5: cyclin dependent kinase 5
CL: cardiolipin
CypD: cyclophilin D
DGAT2: diacylglycerol acyltransferase 2
DRP1: dynamin-related protein 1
ER: endoplasmic reticulum
FIS1: mitochondrial fission 1
FUNDC1: FUN14 domain-containing protein 1
GRP75: 75-kDa glucose-regulated protein
GRP78: 78-kDa glucose-regulated protein
H_2_O_2_: hydrogen peroxide
HDL: high-density lipoprotein
IMM: inner mitochondrial membrane
IP3Rs: inositol 1,4,5-triphosphate receptors
IRE1 α: inositol-requiring protein 1 α
MAM: mitochondria-associated endoplasmic reticulum membranes
MAPK: mitogen-activated protein kinase
MEFs: mouse embryonic fibroblasts
MFN1/2: mitofusin ½
MOSPD2: motile sperm domain-containing protein 2
mPTP: mitochondrial permeability transition pore
mTORC2: mammalian TOR complex 2
OMM: outer mitochondrial membrane
ORP: oxysterol-binding protein-related protein
PA: phosphatidic acid
PC: phosphatidylcholine
PACS-2: phosphofurin acidic cluster sorting protein 2
PDK4: pyruvate dehydrogenase kinase 4
PDZD8: PDZ domain-containing protein 8
PE: phosphatidylethanolamine
PEMT2: phosphatidylethanolamine-*N*-methyltransferase 2
PERK: protein kinase RNA-like endoplasmic reticulum kinase
PG: phosphatidylglycerol
PI: phosphatidylinositol
PML: promyelocytic leukemia protein
PS: phosphatidylserine
PSS: phosphatidylserine synthase
PSD: phosphatidylserine decarboxylase
PP2A: protein phosphatase 2A
PTEN: phosphatase and tensin homolog
PTPIP51: tyrosine phosphatase interacting protein 51
ROS: reactive oxygen species
RyR: ryanodine receptor
SCD1: stearoyl-CoA desaturase 1
SERCA: sarco/endoplasmic reticulum Ca^2+^-ATPase
Sig1R: sigma 1 receptor
StAR: steroidogenic acute regulatory protein
STX17: syntaxin 17
T2DM: type 2 diabetes mellitus
TCHP: trichoplein
TG2: transglutaminase type 2
TOM: translocase of outer mitochondrial membrane
VAPB: vesicle-associated membrane protein-associated protein B
VDAC1: voltage-dependent anion channel 1
WASF3: Wiskott-Aldrich syndrome protein family member 3.
